# Dysphagie nach Extubation auf der Intensivstation: Epidemiologie, klinischer Verlauf und Management

**DOI:** 10.1007/s00063-025-01266-9

**Published:** 2025-04-15

**Authors:** Daniela Bertschi, Jan Waskowski, Philipp Venetz, Carmen A. Pfortmueller, Joerg C. Schefold

**Affiliations:** Universitätsklinik für Intensivmedizin, Inselspital, Universitätsspital Bern, Universität Bern, Freiburgstrasse 18, 3010 Bern, Schweiz

**Keywords:** Schluckstörung, Aspiration, Kritische Erkrankung, Intensivmedizin, Risikofaktoren, Deglutition disorders, Aspiration, Critical illness, Critical care, Risk factors

## Abstract

Schluckstörungen nach Extubation (Postextubationsdysphagie, PED) sind auf Intensivstationen häufig vorhanden und machen etwa 20 % eines gemischt medizinisch-chirurgischen Notfallpatientenkollektivs von Intensivstationen aus. Die PED ist sowohl im Kollektiv neurologischer als auch nichtneurologischen Intensivstationspatienten unabhängiger Risikofaktor für eine erhöhte 28- und 90-Tage-Mortalität (28-Tage-Mortalität: plus 9 %). Das erhöhte Mortalitätsrisiko ist bis zu etwa einem Jahr nach Intensivstationsaufenthalt nachzuweisen. Aufgrund der Konsequenzen von PED sollte bei allen Intensivstationspatienten nach Extubation/Dekanülierung ein systematisches Dysphagiescreening erfolgen (z. B. Wasserschlucktest) und zur Diagnosesicherung eine fiberoptische endoskopische Evaluation des Schluckakts (FEES). Die Behandlung erfolgt interdisziplinär mit Ernährungsanpassung/Nahrungsaufbau bzw. Nahrungskarenz, Physiotherapie/Logopädie und zukünftig ggf. mit interventionellen Verfahren.

## Abstract (en)

Postextubation dysphagia (PED) is common in intensive care units (ICU), affecting about 20% of patients of mixed medical surgical ICU populations. PED is an independent risk factor for increased 28-day and 90-day mortality in both neurological and nonneurological ICU patients (28-day mortality: plus 9%). The increased mortacity risk can be demonstrated for up to approximately one year after the ICU stay. Due to the consequences of PED, all ICU patients should undergo systematic dysphagia screening after extubation/decannulation (e.g., water swallow test) and fiberoptic endoscopic evaluation of the swallowing (FEES) to confirm the diagnosis. Treatment is interdisciplinary with nutrition adaptation/nutrition introduction or food restriction, physical/speech therapy and, if necessary, interventional procedures in the future.

## Lernziele

Nach Lektüre dieses Beitrags …

- können Sie Häufigkeit und Folgen der Postextubationsdysphagie (PED) einschätzen;
- kennen Sie die Risikofaktoren der PED und wissen Sie, dass alle Intensivstationspatienten Risikopatienten („at risk population“) für PED sind;
- ist Ihnen bekannt, dass PED ein unabhängiger Risikofaktor für die 28-Tage- und 90-Tage-Mortalität ist und Effekte auf die Mortalität noch innerhalb eines Jahrs nachweisbar sind;
- kennen Sie die Empfehlung, dass ein systematisches Screening aller Patienten auf Intensivstationen nach Extubation durchgeführt werden sollte;
- wissen Sie, dass ein systematisches Dysphagiescreening direkte Auswirkungen auf das klinische Management betroffener Patienten hat.

## Fallbeispiel

Eine 62-jährige Patientin stürzt im häuslichen Umfeld und zieht sich dabei ein Thoraxtrauma mit Rippenserienfraktur und linksseitiger Lungenkontusion zu. Die Patientin wird initial im regionalen Krankenhaus behandelt und wegen **respiratorischer Insuffizienz**

respiratorischer Insuffizienz

und Agitation intubiert und beatmet. Nach Schmerzeinstellung mit thorakaler Periduralanästhesie (PDA) und Opiaten wird die Patientin nach etwa 48-stündiger Beatmung **extubiert**

extubieren

und von der Intensivstation auf die Normalstation verlegt.

Im Rahmen des dortigen peroralen Kostaufbaus kommt es an Tag 3 nach Extubation anlässlich der erstmaligen Einnahme fester Nahrungsmittel (Brot) zum Erbrechen mit konsekutiver massiver **Aspiration**

Aspiration.

Nach Reintubation erfolgt bei klinischem Verdacht auf Thoraxinstabilität und schwere Oxygenierungsstörung die Verlegung an die Intensivstation des tertiärmedizinischen Zentrums.

In der auf der Intensivstation initial durchgeführten Bronchoskopie wurden feste Nahrungsreste und putrides Sekret abgesaugt. Bei Fieber und linksseitigem Infiltrat in der Röntgenaufnahme des Thorax wird eine empirische antibiotische Therapie eingeleitet. Hierunter verbessert sich die Oxygenierungsstörung innerhalb der nächsten 2 Tage. Der Thorax wird vom Behandlungsteam als stabil beurteilt und die Patientin wird erneut extubiert.

Im Rahmen des systematischen bettseitigen **Dysphagiescreenings**

Dysphagiescreening,

das durch die zuständige geschulte Intensivpflegefachperson erfolgt, wird gemäß institutionellem Standard ein **Wasserschlucktest**

Wasserschlucktest

(WST) durchgeführt. Hierbei kommt es zu heftigen Hustenattacken. In der zeitnah durchgeführten **fiberoptischen endoskopischen Evaluation des Schluckakts**

fiberoptische endoskopische Evaluation des Schluckakts

(FEES) wird die Diagnose einer Schluckstörung gesichert. Auf der **Penetrations-Aspirations-Skala**

Penetrations-Aspirations-Skala

(PAS) zeigt sich ein Score von 7 = Penetration von Flüssigkeit in die Trachea mit insuffizienter Elimination.

Die Patientin wird im weiteren Verlauf bis zur Krankenhausentlassung intensiv physiotherapeutisch betreut. Neben regelmäßigem **Schlucktraining**

Schlucktraining,

allgemeinem Muskelaufbau und respiratorischem Training gelingt nach anfänglicher vollständiger oraler Nahrungskarenz (überbrückend Ernährung über Jejunalsonde) innerhalb der nächsten 2 Wochen der vollständige orale Kostaufbau. Die im Verlauf durchgeführte Kontroll-FEES zeigt einen normalen Schluckakt.

## Hintergrund

Der **Schluckreflex**

Schluckreflex

ist ein bereits in der fetalen Entwicklung etablierter physiologischer (Schutz‑)Reflex, der einerseits eine gerichtete Bewegung des Speisebreis sicherstellt und gleichzeitig den Respirationstrakt vor eindringender Nahrung bzw. entsprechenden Bestandteilen schützt [1]. Physiologisch schluckt ein wacher Mensch täglich etwa 2500-mal, wobei für den physiologischen **Schluckakt**

Schluckakt

das koordinierte Zusammenspiel von etwa 50 Nerven und Muskeln benötigt wird [1]. Im Nervensystem wird der Schluckakt von Schluckzentren im Hirnstamm, höheren suprabulbären und kortikalen Zentren, und den Hirnnerven N. trigeminus, N. facialis, N. glossopharyngeus, N. vagus, und N. hypoglossus gesteuert. Anatomisch sind am Schluckvorgang die Mundhöhle, der Rachen, der Kehlkopf, die Speiseröhre und der Magen beteiligt. Physiologisches Schlucken wird typischerweise in 4 Phasen unterteilt: 1) die orale Vorbereitungsphase, 2) die orale Transportphase, 3) die pharyngeale Phase und 4) die ösophageale Phase [1].

Unsicheres Schlucken bzw. **Schluckstörungen**

Schluckstörungen

(Dysphagie) werden bei kritisch Kranken häufig beobachtet [2]. Auf Intensivstationen sind sie mit teils schwerwiegenden medizinischen Konsequenzen assoziiert [2, 3, 4, 5, 6, 7, 8, 9]. Aus intensivmedizinischer Sicht scheint die Aspiration von Nahrungsbestandteilen in den Respirationstrakt mit nachfolgender „Pneumonie/Pneumonitis“ und/oder resultierender Beatmungspflichtigkeit von besonderer Bedeutung zu sein [2, 3, 4, 5, 6, 7, 8, 9]. Weitere relevante Probleme der Dysphagie umfassen einen verzögerten Kostaufbau bzw. Mangelernährung und/oder Kachexie, verlängerten Aufenthalt auf der Intensivstation bzw. im Krankenhaus, erhöhte Wiederaufnahmeraten auf die Intensivstation, einen höheren intensivmedizinischen Ressourceneinsatz und eine erhöhte 28-Tage- und **90-Tage-Mortalität**

90-Tage-Mortalität

[2].

Wichtig erscheint, dass Dysphagie nicht (wie in älteren Untersuchung teils vermutet) nur in neurologischen, sondern auch in nichtneurologischen Patientenkollektiven von Intensivstationen ein unabhängiger Prädiktor für Tod darstellt [2] und somit alle Intensivstationspatienten als Risikopatienten zu betrachten sind. Die Daten der größten durchgeführten Untersuchung mit systematischem Dysphagiescreening nach Extubation (DYnAMICS-Studie

DYnAMICS-Studie

[2]) zeigen, dass die 28-Tage-Mortalität bei Patienten um etwa plus 9 % erhöht ist [2]. In der Nachverfolgung der Studienpatienten zeigte sich, dass der nachweisbare Effekt auf die Mortalität bis zu etwa einem Jahr nach Intensivstationsaufenthalt nachweisbar ist [10].

Die Dysphagie stellt somit ein relevantes medizinisches Problem dar, das erhebliche Auswirkungen nicht nur auf das betroffene Individuum, sondern auch auf öffentliche Gesundheitssysteme hat [11, 12].

## Diagnostisches Vorgehen

Wie in einer Metaanalyse gezeigt [13] tritt bei einer hohen Anzahl von Patienten nach Extubation eine relevante Schluckstörung auf (zur Inzidenz siehe im Folgenden). Der ideale Zeitpunkt des **Schluckscreenings**

**Abb. 1:**
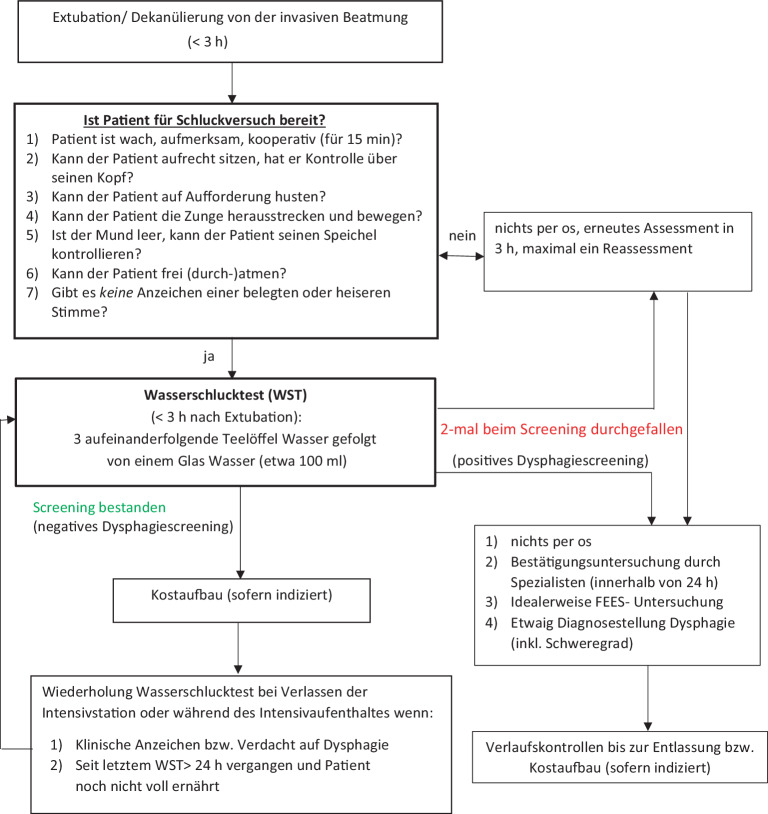
Berner Dysphagiescreeningalgorithmus zur Durchführung auf der Intensivstation. *FEES* fiberoptische endoskopische Evaluation des Schluckakts. [15]

Schluckscreenings

ist wissenschaftlich nicht eindeutig geklärt, meist erfolgt es innerhalb von 24 h nach Extubation. Laut Expertenmeinung [14] sollte das Screening durchgeführt werden, sobald der Patient dazu bereit ist (Abb. 1), idealerweise innerhalb von 8 h nach Extubation.

Die Diagnostik sollte standardisiert anhand internationaler Expertenempfehlungen [14] erfolgen (weitere Literatur in [3, 4, 8, 9, 32]). Dabei wird üblicherweise ein schrittweises **2‑stufiges Vorgehen**

2‑stufiges Vorgehen

empfohlen:

Im ersten Schritt sollte bettseitig ein systematisches Screening durch das Pflegepersonal der Intensivstation erfolgen, wobei die Verwendung eines Tests mit möglichst hoher Sensitivität wichtig ist. Hierbei kann der WST ein **pragmatisches Screeningtool**

pragmatisches Screeningtool

für größere Kollektive von Intensivstationspatienten darstellen. Er ist einfach zu schulen, leicht für Pflegende anzuwenden und wenig zeit- bzw. ressourcenintensiv [2]. Die Durchführung eines systematischen Screenings bei allen Patienten ist entscheidend, da *alle Intensivstationskollektive* betroffen sind. Der WST wird meist mit **progressiver Flüssigkeitsmenge**

progressiver Flüssigkeitsmenge

durchgeführt (z. B. ein Teelöffel bis zu einem Glas [14, 15]). Des Weiteren kann der Test verschiedene Nahrungskonsistenzen beinhalten. Die Beurteilung erfolgt meist durch das geschulte Pflegepersonal und beruht auf Veränderungen der Stimme und respiratorischen Symptomen [16].

Alternativ zum WST-Screening kann auch der komplexere Gugging Swallowing Screen für die Intensivstation (GUSS-ICU) durch entsprechend spezialisiertes Personal durchgeführt werden, der konsekutiv das Schlucken mit verschiedenen Konsistenzen (Trinken einer breiigen, flüssigen, festen, nachfolgend fest-flüssigen Komponente und Wasser) untersucht.

### Diagnosesicherung

Bei Screeningpositivität wird ein **Bestätigungstest**

Bestätigungstest

durch eine spezialisierte (Experten‑)Untersuchung innerhalb von 24 h empfohlen (2-schrittiger Algorithmus). Ziel ist es, im 2. Untersuchungsschritt (**Expertenuntersuchung**

Expertenuntersuchung)

die Diagnose zu bestätigen oder zu verwerfen.

Die Diagnosesicherung bei kritisch kranken Patienten kann mittels 2 unterschiedlicher Verfahren erfolgen: videofluoroskopische Schluckuntersuchung („VFSS“) und FEES. Nachteil der VFSS ist, dass sie nicht auf der Intensivstation am Bett des Patienten durchführbar ist. Daher kann die FEES als diagnostischer Bestätigungstest laut Expertenempfehlung als aktueller **Goldstandard**

**Abb. 2:**
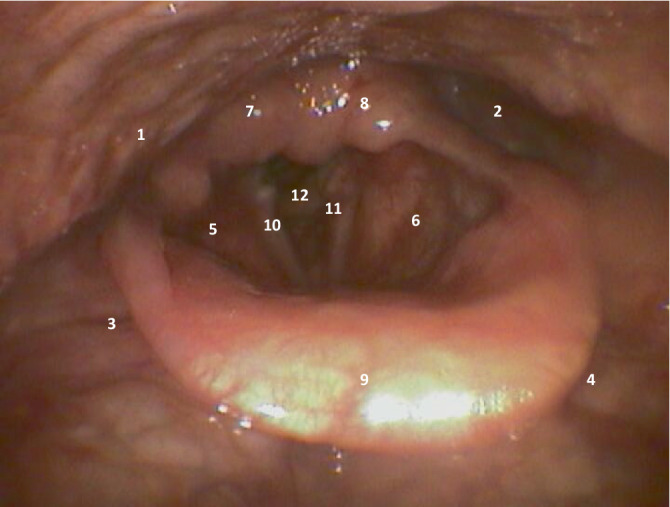
Endoskopische Darstellung des Hypopharynx mit Sinus piriformis (*1,* *2*), Valleculae (*3,* *4*), Aditus laryngis mit Taschenfalten (*5,* *6*), Aryknorpel (*7,* *8*), Larynx mit Epiglottis (*9*), Stimmlippen (*10,* *11*), Trachea (*12*)

Goldstandard

auf Intensivstationen betrachtet werden [14]. Sie ist bettseitig vom trainierten Untersucher durchführbar und somit weder mit einem Patiententransport noch mit einer Strahlenbelastung von Patient und ggf. Untersucher verbunden ([17]; endoskopische Darstellung in Abb. 2).

Das dünne **FEES-Endoskop**

FEES-Endoskop

wird hierbei durch den geschulten Untersucher üblicherweise ohne Betäubung durch die Nase eingeführt und stellt nachfolgend das Schlucken auf einem Monitor dar (typischerweise Anreichung von mit Nahrungsmittelfarbe angefärbtem Wasser oder Nahrung mit gefärbter breiiger Konsistenz).

Diese funktionelle Untersuchung erlaubt durch eine direkte Beobachtung des Schluckakts eine **Graduierung**

Graduierung

entsprechend der Erkrankungsschwere. Der Vorteil dieser Untersuchung ist, dass sie mit wenig apparativem und personellem (Ressourcen‑)Aufwand jederzeit direkt am Patientenbett durchgeführt werden kann und für den Patienten nicht mit einer Strahlenbelastung und insgesamt mit geringen Risiken verbunden ist.

#### Merke

Laut Expertenempfehlung stellt aktuell die FEES-Untersuchung den Goldstandard als diagnostischen Bestätigungstest dar.

### Beispielhafter bettseitiger Screeningalgorithmus

Ein Beispiel eines bettseitigen Screeningalgorithmus ist der von den Autoren vorgeschlagene „Berner Dysphagiescreeningalgorithmus, BIDA“ ([15]; Abb. 1, Grundlage ist der Wasserschlucktest mit akzeptabler Sensitivität/ Spezifität; formale Validierung ausstehend). Dieser Algorithmus sieht vor, dass alle Patienten ohne Ausschlusskriterien (i.e. finale Extubation) innerhalb der ersten 3 h nach Extubation oder Dekanülierung im Sinne eines **„Sicherheitschecks“**

„Sicherheitschecks“

überprüft werden, ob sie die genannten Grundvoraussetzungen für einen WST erfüllen. Hiernach werden 3‑mal ein Teelöffel und anschließend ein Glas Wasser zum Schlucken verabreicht und die betreuende geschulte Pflegefachperson beurteilt, ob klinische Anhaltspunkte für eine Dysphagie vorliegen. Bei positivem Screening werden die Patienten für einen diagnostischen Bestätigungstest und entsprechendes Schlucktraining der **Physiotherapie**

Physiotherapie

zugeführt. Es erfolgt, wenn immer möglich, eine instrumentelle Bestätigungsuntersuchung mittels FEES.

## Schweregrade

Das Ergebnis der FEES wird üblicherweise mittels PAS graduiert (Tab. 1). Der **PAS-Score**

**Abb. 3:**
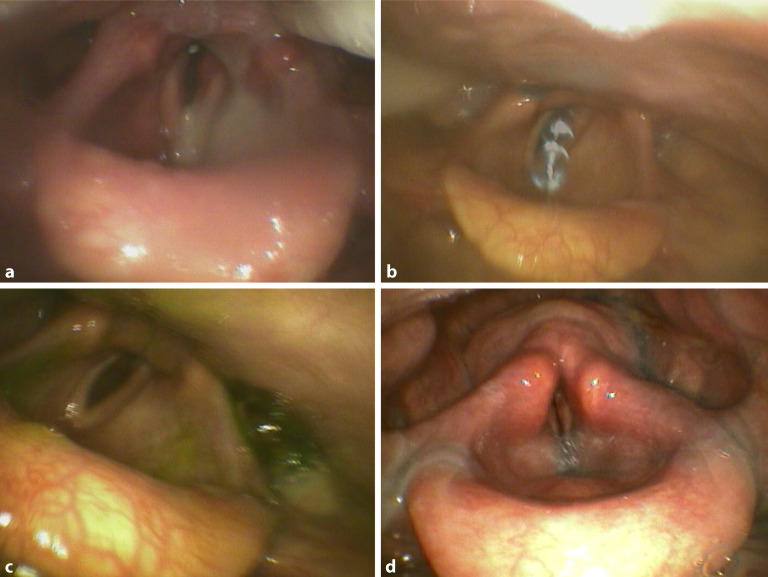
Pathologische endoskopische Befunde. **a** Spontane Aspiration von eitrigem Sekret. **b** Schlucken mit direkter Aspiration. **c** Penetration in Aditus. **d** Aspiration in Trachea

PAS-Score

bewertet hierbei, wie tief Material in die Luftwege eindringt (bis oberhalb, bis auf Höhe, bis unterhalb der Stimmbänder), ob Material in den Luftwegen verbleibt und wie kräftig der Patient das Material mittels Hustenstoß wieder entfernen kann [18, 19]. Das Scoring lässt sich sowohl bei der VFSS [18] als auch insbesondere bei der FEES [17, 20] anwenden (Abb. 3).

**Tab. 1:** Penetrations-Aspirations-Skala (*PAS*) [19]

Score	Charakteristika
PAS 1	Material dringt nicht in die Luftwege ein
PAS 2	Material dringt in die Luftwege ein, verbleibt oberhalb der Stimmlippen und wird anschließend aus den Luftwegen entfernt
PAS 3	Material dringt in die Luftwege ein, verbleibt oberhalb der Stimmlippen und wird anschließend trotz Hustenstoß nicht komplett aus den Luftwegen entfernt
PAS 4	Material dringt in die Luftwege ein, berührt die Stimmlippen und wird anschließend aus den Luftwegen entfernt
PAS 5	Material dringt in die Luftwege ein, berührt die Stimmlippen und wird anschließend trotz Hustenstoß nicht komplett aus den Luftwegen entfernt
PAS 6	Material dringt in die Luftwege bis unterhalb der Stimmlippen ein, wird anschließend aber aus den Luftwegen entfernt
PAS 7	Material dringt in die Luftwege bis unterhalb der Stimmlippen ein und wird anschließend trotz Hustenstoß nicht komplett aus den Luftwegen entfernt
PAS 8	Material dringt in die Luftwege bis unterhalb der Stimmlippen ein, es wird keine Anstrengung unternommen, das Material aus den Luftwegen zu entfernen

Wenig Evidenz existiert jedoch für die Beurteilung, welcher exakte Schweregrad einer mittels FEES diagnostizierten Dysphagie für den Patienten bezüglich patientenbezogener Endpunkte [21] relevant ist. Es ist anzunehmen, dass die **Komplikationsrate**

Komplikationsrate

zunimmt, je tiefer und ausgiebiger die Penetration in die Luftwege erfolgt. Analog hierzu wurde der 4‑stufige „FEES dysphagia score“ [20] entwickelt, der aktuell vor allem bei neurologischen Patienten verwendet wird. Derzeit (vor allem im Kollektiv von Parkinson-Patienten) validiert ist zudem die Dynamic-imaging-grade-of-swallowing-toxicity(DIGEST)-FEES-Skala [21], eine 5‑stufige Skala, die zusätzlich zur PAS-Bewertung jedes einzelnen Bolusschlucks die Häufigkeit und Quantität der Aspirationen berücksichtigt und damit **Schluckeffizienz**

Schluckeffizienz

und Schlucksicherheit präziser bewertet [20]. Inwieweit jedoch verschiedene Scores bzw. klinische Testverfahren für die exakte (Verlaufs‑)Beurteilung und genaue Graduierung der PED im gemischten Kollektiv von kritisch kranken Patienten anwendbar sind [22], bleibt abzuwarten.

## Inzidenz

In der größten zur Inzidenz der Dysphagie auf Intensivstationen prospektiv durchgeführten Studie („DYnAMICS“, *n* = 1304) mit systematischem Dysphagiescreening wird die Häufigkeit der PED im gemischten medizinisch-chirurgischen und notfallmäßig auf die Intensivstation zugewiesenen erwachsenen Patientenkollektiv mit 18,3 % angegeben [2]. Die PED-Inzidenz bei elektiven postoperativen Patienten beträgt etwa 5 % [2]. Zuvor wurde die Inzidenz der Dysphagie bei kritisch Kranken in der Literatur mit „3–62 %“ benannt [9], was auf eine initial unsichere **Datenlage**

Datenlage

hinwies (dies vermutlich aufgrund hoher Heterogenität der kleinen Patientenkollektive, in denen kein systematisches Dysphagiescreening erfolgte). Die PED-Inzidenz zum Zeitpunkt der Entlassung von der Intensivstation beträgt 10,3 % und eine Mehrzahl der Patienten (60,4 %), die die Intensivstation mit Dysphagie verlassen, zeigen in der physiotherapeutischen Nachbetreuung bis zur Krankenhausentlassung weiterhin eine Dysphagie [2]. Studien bei Patienten mit „acute respiratory distress syndrome“ (ARDS) wiesen nach, dass eine Dysphagie bis zu mehreren Jahren und damit weit über die Krankenhausentlassung hinaus bestehen kann [16]. Aktuell ist jedoch die Datenlage zum Verlauf von Intensivstationspatienten, die mit Dysphagie das Krankenhaus verlassen, limitiert und weitere epidemiologische Studien erscheinen nötig.

## Risikofaktoren

Die beschriebenen Risikofaktoren der PED sind: 1) akute neurologische Erkrankung, 2) Notfallzuweisung auf die Intensivstation, 3) Dauer der invasiven mechanischen Beatmung bzw. 4) Tage mit Nierenersatztherapie und 5) erhöhte Erkrankungsschwere (erhöhter Acute-physiologic-assessment-and-chronic-health-evaluation[APACHE]-II-Score; [23]). Als typischer **Hochrisikopatient**

Hochrisikopatient

für eine PED kann ein erwachsener kritisch kranker Patient gelten, der aufgrund einer akuten neurologischen Erkrankung als Notfall auf die Intensivstation aufgenommen wird und über mehrere Tage beatmungspflichtig ist [2, 23, 24, 25, 26].

## Effekte auf die Mortalität

Nach Adjustierung für typische Confounder ist PED ein unabhängiger **Prädiktor**

Prädiktor

für die 28-Tage- und 90-Tage-Mortalität (die zusätzliche 90-Tage-Mortalität beträgt plus 9,2 %, *n* = 116; [2]). Das multivariate Risiko (Hazard-Ratio, HR) für eine 90-Tage-Mortalität beträgt in der DYnAMICS-Studie 2,95 (95 %-Konfidenzintervall [95 %-KI] 1,57–5,53, *p* < 0,001; [2]). Effekte auf die Mortalität lassen sich in der **Langzeitnachverfolgung**

Langzeitnachverfolgung

(*n* = 273; maximale Beobachtungsdauer: 6 Jahre; durchschnittliche Nachverfolgung 4,7 ± 2,1 Jahre) bis etwa ein Jahr (360-Tage-HR: 1,03; 95 %-KI 0,42–3,70) nach Entlassung von der Intensivstation nachweisen: In Dysphagie-screening positiven (vs. -negativen) Patienten ist die 180-Tage-Mortalität 16 % vs. 5,8 % (zusätzliche Mortalität plus 10,2 %) und die 360-Tage-Mortalität 25 % vs. 9,1 % (zusätzliche Mortalität plus 15,9 %; [10]). Die mittlere HR bezogen auf die Mortalität im ersten Jahr nach Aufnahme auf die Intensivstation betrug 2,09 (95 %-KI, 1,34–3,24; *p* = 0,0009; [10]).

### Merke

Wichtig erscheint, dass Dysphagie sowohl im Kollektiv neurologischer als auch nichtneurologischer Intensivstationspatienten ein unabhängiger Risikofaktor für eine erhöhte 28-Tage- und 90-Tage-Mortalität ist und alle Patientengruppen innerhalb der Intensivmedizin nach Extubation oder Dekanülierung betrifft [2].

## Ätiologie

Die zugrunde liegende exakte Pathophysiologie der PED bzw. entsprechende pathophysiologische Wege zur Entwicklung der PED bleiben aktuell unklar. In der Literatur werden 6 potenzielle primäre Pathomechanismen postuliert bzw. diskutiert [1, 3, 4]:

### Laryngeales Trauma

Erstens kann PED durch ein *direktes oropharyngeales und laryngeales Trauma im Rahmen einer (ggf. notfallmäßig erfolgten) Intubation entstehen und/oder durch den zuvor in situ liegenden Trachealtubus* hervorgerufen bzw. unterhalten werden. Selbiges gilt für andere Sonden im Pharynxbereich wie z. B. Sonden zur transösophagealen Echokardiographie (TEE; [27, 28]). Anatomisch betrachtet können u. a. **Druckschäden**

Druckschäden,

Ulzerationen oder Vernarbungen im Bereich der Stimmbänder auftreten [4]. Entsprechende lokale Ulzerationen und/oder Druckschäden oder Entzündungen können die Stimmbänder, die Epiglottis, die Aryknorpel oder die Zunge/den Zungengrund beschädigen, wodurch die Schutzfunktion dieser anatomischen Strukturen gegen Aspiration beeinträchtigt werden kann. Ferner kann es durch Dislokationen bzw. Subluxation der Aryknorpel zu **vermindertem Glottisschluss**

vermindertem Glottisschluss

während des Schluckakts kommen. Ebenfalls ist denkbar, dass aufgrund einer Kompression des N. laryngeus recurrens Stimmbandlähmungen auftreten. Zuletzt können Lippen- und Zahnverletzungen sowie orale Schwellungen zu Störungen der oralen Phase des Schluckakts führen [4].

### Neuromuskuläre Dysfunktion

Zweitens wird PED im Sinne einer erworbenen *neuromuskulären Schwäche *(in der Literatur auch als „intensive care unit-acquired swallowing disorder, ICU-ASD“ erwähnt) interpretiert. Eine neuromuskuläre Schwäche kann durch/mit Atrophie der Zungen‑, Larynx- und Pharynxmuskulatur z. B. im Rahmen der Intensivtherapie mit prolongierter Intubation und Sedativa- und Relaxanziengabe und ebenso durch die mit der Intensivbehandlung assoziierte generalisierte „critical illness polyneuropathy“ (CIP; [19, 20, 21]) bzw. „critical illness myopathy“ (CIM; [29, 30, 31]) entstehen. Dies kann insbesondere bei systemischen entzündlichen Prozessen (Sepsis, septischer Schock) zur Entwicklung einer generalisierten **neuromuskulären Schwäche**

neuromuskulären Schwäche

führen. Dabei kann die PED als lokale Konsequenz der erwähnten erworbenen neuromuskulären Dysfunktion vergleichbar einer ventilatorinduzierten diaphragmalen Dysfunktion („ventilator-induced diaphragmatic dysfunction“, VIDD), die lokal auf diaphragmaler Ebene entsteht, verstanden werden. Aus klinischer Sicht ist relevant, dass ein gemeinsames Auftreten von PED und VIDD mit vermindertem Hustenstoß und somit eingeschränkter Schutzfunktion auf laryngealer Ebene die PED verstärken kann [1, 4].

### Globale Beeinträchtigung der Wahrnehmung

Viertens kann eine *globale Beeinträchtigung der Wahrnehmung* zugrunde liegen. Diese kann u. a. durch ein **Delir**

Delir,

die intensivmedizinische (kritische) Grunderkrankung oder iatrogen medikamentös induziert werden. Im Fall von neurologischen Krankheitsbildern, wie bei Schädelhirntrauma, Schlaganfall, intrakraniellen Blutungen und/oder entzündlichen Erkrankungen des Zentralnervensystems (ZNS), kann eine PED teilweise auch durch die direkte Schädigung im Bereich des ZNS erklärt werden. Hierbei bleibt zu berücksichtigen, dass beim Delir eine qualitative Bewusstseinsstörung vorliegt und **sedierende Medikamente**

sedierende Medikamente

das Schlucken auf zentraler Ebene im Sinne einer Vigilanzminderung beeinflussen können. Weiterhin ist denkbar, dass peripher wirkende neurotrope Medikamente die neuromuskuläre Übertragung beeinträchtigen können, worin eine Überlappung zur „neuromuskulären Dysfunktion“ bestehen kann [1, 4].

### Gestörte Sensorik

Drittens ist eine intakte Sensorik im Pharynx für einen physiologischen Schluckprozess essenziell [20]. Somit kann die PED potenziell durch eine *dysfunktionale oropharyngeale und laryngeale Sensorik* ausgelöst werden. Die zugrunde liegende Pathophysiologie scheint hier eine Schädigung von diversen afferenten sensorischen Fasern zu sein [1], die sowohl durch die bereits zuvor angesprochene CIP als auch durch ein **lokales Ödem**

lokales Ödem

oder ein direktes lokales Trauma hervorgerufen werden kann. In der Summe führt dies zu einer gestörten zeitlichen Koordination und einer verminderten Stärke des Larynxschlussreflexes. Klinisch kann sich diese Störung insbesondere dann zeigen, wenn ein Nahrungsbolus die „Reflextriggerzone“ am Palatoglossusbogen erreicht: Sofern die entsprechenden afferenten Bahnen geschädigt sind, resultiert hieraus eine verzögerte Schluckantwort, was eine **prädeglutitive Aspiration**

prädeglutitive Aspiration

bedingen kann [1, 4].

### Gastroösophagealer Reflux

Fünftens wird *gastroösophagealer Reflux* ursächlich für die Entwicklung einer PED diskutiert bzw. kann diese unterstützen. Der gastroösophageale Reflux kann durch die liegende Position im Bett sowie Sedation/**Muskelrelaxanzien**

Muskelrelaxanzien

bei intubierten Intensivstationspatienten verstärkt werden – und diese Probleme können sich zeitlich teilweise bis in die Postextubationsphase fortsetzen. Hinzu kommt eine im Rahmen der kritischen Erkrankung oft erworbene Magenentleerungsstörung mit der Notwendigkeit der kontinuierlichen Sondenernährung [1, 4].

### Asynchrones Atmen und Schlucken

Sechstens wird ätiologisch für eine PED *asynchrones Atmen und Schlucken* angenommen. Dieses tritt vor allem bei Patienten mit Tachypnoe und respiratorischen Problemen auf. Physiologischerweise gewährleistet die exakte Koordination von Larynxschluss, Apnoe und Öffnung des oberen Ösophagusspinkters einen effizienten Aspirationsschutz [4]. Bei steigender Atemfrequenz wird die apnoeische Phase im Schluckakt verkürzt, wodurch häufiger Aspirationen auftreten können [1, 4].

### Resümee

Zusammengefasst handelt es sich ätiologisch bei PED vermutlich häufig um eine klinisch relevante erworbene neuromuskuläre Störung auf Kehlkopfebene, wobei verschiedene Faktoren ursächlich beitragen können.

#### Merke

Ätiologisch handelt es sich bei PED vermutlich häufig um eine klinisch relevante erworbene neuromuskuläre Störung auf Kehlkopfebene, wobei verschiedene Faktoren ursächlich beitragen können.

Interessant erscheint, dass PED auch in Patientenkollektiven auftritt, in denen man dies nicht per se vermuten würde. Beispielsweise entwickeln etwa 5 % der Patienten nach elektiven meist kurzen Operationen (unkomplizierte Kardiochirurgie o. ä.) eine PED [2]. Wissenschaftlich könnte eine vertiefte Analyse dieser Population für ein verbessertes Verständnis der PED-Pathophysiologie besonders interessant sein.

## Therapeutische Optionen

Die Therapie der PED besteht aktuell aus 3 wesentlichen Komponenten: 1) adaptive Maßnahmen, 2) kompensatorische Behandlung bzw. 3) funktionelle Übungen [33].

Zunächst kann eine *Anpassung der Nahrung bzw. der ***Nahrungskonsistenz**

Nahrungskonsistenz

erfolgen. Hierbei ist jedoch zu bemerken, dass aktuell keine gute Evidenz [34] existiert, dass die entsprechende Therapie Einfluss auf harte klinische Endpunkte bei kritisch Kranken hat. Die zusätzlichen Komponenten umfassen **Kompensationsstrategien**

Kompensationsstrategien,

worunter einerseits spezielle Schlucktechniken und andererseits die *Einnahme spezieller Körperhaltungen* verstanden werden, mit dem Ziel, Schluckstörungen auszugleichen [1, 20, 35]. Die zu erlernenden *Schlucktechniken* zielen hierbei vor allem auf Patienten ab, bei denen der Schluckreflex verzögert ist oder anatomische Veränderungen vorhanden sind (z. B. nach Stimmbandoperationen). Obwohl für Schlaganfallpatienten die Effizienz dieser Interventionen (siehe auch Labeit et al. [35]) belegt ist, gibt es für kritisch kranke Patienten auf Intensivstationen hierzu nur beschränkte Evidenz.

Ferner wurde in den letzten Jahren die **neuromuskuläre Elektrostimulation**

neuromuskuläre Elektrostimulation

auf kranialer und auf pharyngealer Ebene getestet [35], wobei Daten vor allem bei Patienten mit Schlaganfall existieren [28, 35]. So zeigen z. B. die Daten der PHAST-TRAC-Studie mit tracheotomierten Patienten den Nutzen der Elektrostimulation bezüglich Dekanülierung (Odds-Ratio [OR] 7,0; 95 %-KI 2,41–19,88, *p* = 0,0008; [36]). Die Wirksamkeit der neuromuskulären Stimulation bei PED, d. h. bei kritisch kranken Patienten auf Intensivstationen, ist derzeit Gegenstand einer laufenden multizentrischen Studie [37]. Die pharyngeale Elektrostimulation (PES) wird bei definierten Patienten mit neurogener Dysphagie (z. B. nach Schlaganfall [38]) empfohlen.

## Fazit für die Praxis

- Die Postextubationsdysphagie (PED) ist häufig und ein unabhängiger Risikofaktor für Mortalität.
- Aufgrund von Häufigkeit, assoziierten Komplikationen (u. a. Aspiration, Malnutrition) und vorhandenen Behandlungsoptionen sollten alle Intensivstationspatienten nach Extubation/Dekanülierung einem systematischen PED-Screening zugeführt werden (z. B. per Wasserschlucktest).
- Bei Intensivstationspatienten mit positivem PED-Screening sollte eine fiberoptische endoskopische Evaluation des Schluckakts (FEES) zur Diagnosesicherung erfolgen.
- Publizierte 2‑stufige systematische Screeningabläufe (z. B. Berner Dysphagiescreeningalgorithmus BIDA, entwickelt zur Durchführung auf der Intensivstation) unterstützen einen fokussierten Ressourceneinsatz.
- Obwohl die PED-Pathophysiologie derzeit noch unklar ist, kann davon ausgegangen werden, dass eine erworbene neuromuskuläre Störung vorliegt.
- Die Behandlung der PED sollte im interdisziplinären Team erfolgen (Pflege, Arzt, Physiotherapie/Logopädie, ggf. HNO).

## CME-Fragebogen

Wie häufig ist Dysphagie nach Extubation in einem gemischt medizinisch-chirurgischem Kollektiv erwachsener Intensivstationspatienten mit initialer notfallmäßiger Aufnahme?

Etwa 5 %

Etwa 10 %

Etwa 20 %

Etwa 30 %

Etwa 40 %?

Wie häufig ist Dysphagie nach Extubation in einer elektiv postchirurgisch (z. B. nach Kardiochirurgie) auf die Intensivstation aufgenommenen Patientenklientel?

Etwa 5 %

Etwa 10 %

Etwa 20 %

Etwa 30 %

Etwa 40 %

Was wird aktuell meist als wichtigster ätiologischer Faktor für die Entwicklung einer intensivtherapieassoziierten erworbenen Postextubationsdysphagie (PED) vermutet?

Erworbene neuromuskuläre Dysfunktion

Initiale hypertone Entgleisung

Folgen einer disseminierten intervasalen Gerinnungsstörung

Bislang nicht diagnostizierte Vaskulitis

Persistierende Sympathikusaktivierung

Wann sollte immer eine (erste) Screeninguntersuchung auf Postextubationsdyspghagie (PED) sinnvollerweise auf der Intensivstation durchgeführt werden?

Bei Aufnahme auf die Intensivstation

Nach Entlassung von der Intensivstation

Nach Extubation oder Dekanülierung

Vor Entlassung aus dem Krankenhaus

Nach beobachteter Aspiration

Alle Intensivstationspatienten haben das Risiko für die Entwicklung einer Postextubationsdysphagie (PED). Welcher der folgenden (erwachsenen) Patienten hat ein besonders hohes Risikoprofil für eine PED?

Patient nach notfallmäßiger Aufnahme auf die Intensivstation aufgrund akuter neurologischer Erkrankung und Beatmungspflicht für 5 Tage

Patient mit schwerer allergischer Reaktion (Grad 2 zum Zeitpunkt der Intensivstationsaufnahme) aufgrund Prick-Testung in der Dermatologie

Patient mit „acute kidney injury“ mit Oligurie, derzeit in der Phase spontaner renaler Erholung

Patient nach komplexer Pankreaschirurgie bei Pankreaskarzinom

Patient nach Polytrauma mit beidseitiger Lungenkontusion

Ein 45-jähriger Patient war aufgrund eines kardiogenen Schocks (STEMI) während 5 Tagen intubiert und wurde am Vormittag extubiert. Welche Untersuchung ist der Goldstandard für die Bestätigung einer vermuteten Dysphagie?

Breischluck (Videofluoroskopie)

Fiberoptische endoskopische Evaluation des Schluckakts (FEES)

Ösophagogastroduodenoskopie (ÖGD)

Computertomographie (CT)

Wasserschlucktest (WST)

Was bedeutet ein Penetrations-Aspirations-Skala(PAS)-Wert von 1 in der durchgeführten fiberoptischen endoskopischen Untersuchung des Schluckakts (FEES)?

Material dringt nicht in die Luftwege ein.

Material dringt in die Luftwege ein, berührt die Stimmlippen und wird anschließend aus den Luftwegen entfernt.

Material dringt in die Luftwege bis unterhalb der Stimmlippen ein, wird anschließend aber aus den Luftwegen entfernt.

Material dringt in die Luftwege ein, verbleibt oberhalb der Stimmlippen und wird anschließend trotz Hustenstoß nicht komplett aus den Luftwegen entfernt.

Material dringt in die Luftwege bis unterhalb der Stimmlippen ein, es wird keine Anstrengung unternommen, das Material aus den Luftwegen zu entfernen.

In einer durchgeführten fiberoptischen Untersuchung des Schluckakts (FEES) wird ein Penetrations-Aspirations-Skala(PAS)-Score von 7 festgestellt. Was bedeutet dies?

Material dringt in die Luftwege ein, berührt die Stimmlippen und wird anschließend aus den Luftwegen entfernt.

Material dringt in die Luftwege bis unterhalb der Stimmlippen ein und wird anschließend trotz Hustenstoß nicht komplett aus den Luftwegen entfernt.

Material dringt in die Luftwege ein, verbleibt oberhalb der Stimmlippen und wird anschließend aus den Luftwegen entfernt.

Material dringt in die Luftwege bis unterhalb der Stimmlippen ein, es wird keine Anstrengung unternommen, das Material aus den Luftwegen zu entfernen.

Material dringt in die Luftwege bis unterhalb der Stimmlippen ein, wird anschließend aber aus den Luftwegen entfernt.

Was ist ein etabliertes Therapiekonzept zur Dysphagiebehandlung?

Ernährung über perkutane endoskopische Gastrostomie (PEG)

Schlucktraining

Muskelrelaxation

Transthorakale Elektrostimulation

Gabe von Parasympathomimetika

Was ist ein etabliertes Therapiekonzept bei ausgewählten Patienten mit neurogener Dysphagie (z. B. nach Schlaganfall)?

Applikation von Lokalanästhetika

Pharyngeale Elektrostimulation

Vollheparinisierung für 14 Tage

Orale Gabe von Sympathomimetika

Intravenöse Gabe von Anticholinergika
